# Assessment of sarcopenia in patients with fibromyalgia

**DOI:** 10.1007/s00296-021-04973-6

**Published:** 2021-08-21

**Authors:** Abeline Kapuczinski, Muhammad S. Soyfoo, Sandra De Breucker, Joëlle Margaux

**Affiliations:** 1 Department of Rheumatology, Hôpital Erasme, Université Libre de Bruxelles, 808, Route de Lennik, 1070 Bruxelles, Belgium; 2 Department of Geriatrics, Hôpital Erasme, Université Libre de Bruxelles, Bruxelles, Belgium

**Keywords:** Fibromyalgia, Muscle mass, Muscle strength, Physical performance, Sarcopenia

## Abstract

Fibromyalgia is a chronic disorder characterized by persistent widespread musculoskeletal pain. Patients with fibromyalgia have reduced physical activity and increased sedentary rate. The age-associated reduction of skeletal muscle mass and function is called sarcopenia. The European Working Group on Sarcopenia in Older People developed a practical clinical definition and consensus diagnostic criteria for sarcopenia. Loss of muscle function is common in fibromyalgia and in the elderly. The goal of this study is to determine whether the reduction of muscle function in fibromyalgia is related to sarcopenia according to the European Working Group on Sarcopenia in Older People criteria. Forty-five patients with fibromyalgia and thirty-nine healthy control female subjects were included. All the participants were assessed by Fibromyalgia Impact Questionnaire and SARC-F questionnaire. Muscle mass was evaluated by bioimpedance analysis, muscle strength by handgrip strength test and physical performance with the Short Physical Performance Battery. Fibromyalgia Impact Questionnaire and SARC-F scores were statistically significantly higher in the fibromyalgia group than in the control group, showing severe disease and a higher risk of sarcopenia in the fibromyalgia group (*p* < 0.001). Muscle strength and physical performance were statistically significantly lower in the group with fibromyalgia than in the control group (*p* < 0.001). There was no statistical difference between fibromyalgia and control groups regarding skeletal muscle mass (*p* = 0.263). Our study demonstrated a significant reduction in muscle function in fibromyalgia patients without any loss of muscle mass. Loss of muscle function without decrease in muscle mass is called dynapenia.

**Supplementary Information:**

The online version contains supplementary material available at 10.1007/s00296-021-04973-6.

## Introduction

Fibromyalgia (FM) is a chronic disorder characterized by persistent widespread musculoskeletal pain. It is associated with fatigue, sleep and cognitive disorders, anxiety and depression, leading to a poor quality of life [1]. It affects 2% of the general population and more particularly women [2, 3]. The pathophysiology of FM is complex with several intricating factors. One of the main tenants underlying the disease is a somatosensory disturbance resulting in hypersensitization to pain (allodynia and hyperalgesia) [4]. The diagnosis of FM is based according to the 1990 and 2010 American College of Rheumatology (ACR) diagnostic criteria [5, 6] as well as the updated criteria from 2016 [7]. There is currently no specific treatment of FM, the mainstay of treatment consisting primarily of pain management and physical rehabilitation.

As such, due to widespread pain, patients with FM have a reduced physical activity and a higher sedentary rate [8]. There are several converging lines of evidence demonstrating a significant reduction of muscular mass and physical activity and performance in patients with FM when compared with healthy controls matched for age and sex [9, 10]. Furthermore, FM patients have a significantly increased muscular fat proportion compared to controls [11, 12]. The resulting loss of muscular mass also known as sarcopenia is associated with an increased risk of falls and hence bone fractures [13].

In 2010, the European Working Group On Sarcopenia in Older People (EWGSOP) published a consensus definition of sarcopenia with diagnostic criteria for clinical practice [14]. These criteria were defined by including muscular mass as well as muscular function, strength and physical performance. In 2018, these criteria were updated with the definition of loss of muscular strength as the primary feature of sarcopenia. Sarcopenia was defined as probable if there was a loss of muscle strength and confirmed if the loss of strength was associated with reduced muscle mass. When poor physical performance is also detected, sarcopenia is considered severe [15].

Some studies have compared the physical function and condition of patients with FM to those of elderly patients and have showed that the loss of muscular strength and physical performance were not different [10, 16]. Besides this, there was an increased risk of falls and loss of autonomy in FM patients similarly to that described in older population [13].

The aim of this study was to determine whether the reduction of muscle function in FM is related to sarcopenia according to the EWGSOP 2010 criteria.

## Patients and methods

### Population

The study was approved by the Ethics Committee of Erasme Hospital, with the reference SRB_201710_067 in January 2018. All the participants were informed about the objectives of the study and an informed consent was obtained. This study included two groups: a fibromyalgia group and a healthy control group. The recruitment of fibromyalgia subjects was done from the outpatient clinic from the Department of Rheumatology at Erasme Hospital. The healthy control group consisted of subjects among health care workers and other medical staff from Erasme Hospital on a voluntary basis. The inclusion criteria were female subjects, aged between 30 and 60 years and with a body mass index (BMI) ≤ 30 kg/m^2^. Pregnant women and patients carrying a pacemaker (which are contraindications for using a bio-electrical impedance analysis) were excluded from the study.

Forty-five patients with fibromyalgia, according to the 2010 ACR criteria, were included as well as 39 healthy control females. Patients in FM group did not have other chronic diseases nor other rheumatologic diseases. Anthropometrics measures (age, height, weight and BMI) of the patients were determined. We used the same measuring rod and the same scale for each evaluation. The patients were weighted with an empty stomach, preferably in the morning, with clothes and without shoes.

### Questionnaires

Each patient or control filled the Fibromyalgia Impact Questionnaire (FIQ) of the fibromyalgia and the SARC-F questionnaire of the sarcopenia.

The FIQ assesses the impact of fibromyalgia on patients’ activities of daily life, pain, fatigue, anxiety, depression [17]. It establishes a total score of 100 points, determining severe (> 59/100) or moderate (between 39 and 59/100) disease. The SARC-F questionnaire was developed to screen sarcopenia [18]. It includes five questions about strength, walking ability, stair climbing, rising from a chair and history of falls, giving a score ranging from 0 to 10. A score above or equal to 4 points is predictive of sarcopenia [19].

### Assessment of muscle mass

Muscle mass was quantified with a bio-electrical impedance analysis (BIA) which gives the volume of fat and lean body mass based on the relationship between the volume of a conductor and its electrical resistance [18]. We used the Bodystat QuadScan 4000 (Bodystat ltd, UK) to assess the fat mass content (kg), the lean mass (kg) and resistance at 50 kHz (Ohms). The predicted skeletal muscle mass (SMM) was calculated using the equation of Janssen for the BIA [20]:

$$\begin{array}{*{20}c} {SMM \, = \, \left( {height^{2} /BIA \, resistance*0.401} \right) \, + \left( {gender*3.825} \right) \, + \, \left( {age* - 0.071} \right) \, + \, 5.102),} \\ {with \, height \, in \, centimeters, \, BIA \, resistance \, in \, Ohms, \, gender \, = \, 1\,for \, male \, and \, 0 \, for \, female, \, age \, in \, years.} \\ \end{array}$$

Skeletal muscle mass index (SMI) was obtained by dividing SMM by height squared (m^2^). We compared the results in the two groups with cut-offs defined by the EWGSOP where low muscle mass assessed by BIA is defined by a SMI below 6.42 kg/m^2^ for women.

### Assessment of muscle strength

Muscle strength was evaluated by a handgrip strength test with the Jamar dynamometer. Three measures for each arm have been taken and the best result for the dominant hand has been used for our study. Low muscle strength is characterized by a handgrip strength test below 20 kg for women.

### Assessment of physical performance

Physical performance was assessed with the Short Physical Performance Battery (SPPB). It is a composite test of usual gait speed (over 4 m), a balance test and a chair stand test. The scores range from 0 to 12 points: low performance (0–6 points), intermediate (7–9 points), high performance (10–12 points) [21].

### Assessment of sarcopenia

The European Working Group on Sarcopenia in Older People (EWGSOP) developed in 2010 a practical clinical definition and consensus diagnostic criteria for sarcopenia with threshold values for geriatric populations. We used those values to assess all the participants for the muscle mass, muscle strength and physical performance [14]. According to the EWGSOP, the cut-off for sarcopenia is defined by values of predicted muscle mass lower than 6,42 kg/m^2^. A low muscle strength was considered when the grip strength test was below 20 kg and a low physical performance when the SPPB score was below 8 points.

### Statistics

One propensity score will be performed on two groups: Fibromyalgia (*n* = 45) and Control (*n* = 39). The CBPS R package will be used to perform the propensity score, estimating an Average Treatment Effect (ATE), using covariate balancing and requesting an exact match, which has been showed to be superior to traditional logistic regression approaches and boosted classification and regression trees [22]. An absolute standardized difference less than 10–15% will be considered to support the assumption of balance between the groups because it is not affected by the sample size, unlike P-values, and it may be used to compare the relative balance of variables measured in different units [23]. The mean and standard deviation obtained after matching for continuous variables will be presented. After the propensity score, we will use the survey R package to perform linear regressions for continuous outcomes, which will include the treatment group effect, the weight resulting from the matching and variables present in the propensity score to obtain a doubly robust estimator which will correct the last remaining possible imbalance between the covariates and produces an unbiased treatment effect [29]. The survey R package includes the Huber–White-corrected standard errors, which maintains the standard errors unbiased even under heterogeneity of the residuals [30]. Last, the advantage of a doubly robust estimator is that it needs only one of the two models (propensity score and linear regression after the propensity score) to be correctly specified. The R software (R Core Team, 2017), version 3.4.3. was used to produce the results.

Before drawing conclusions on the above table, we have to apply a Bonferroni correction for multiple comparison purposes and we have to divide the p-value (0.05) by the number of comparisons [9], that is, 0.05/9 = 0.0056, to get the final p-value on which we can draw conclusion. That is, any p-value below 0.0056 will be considered as significant: the next variables are significantly different between the two groups: FIQ, SARC-F, handgrip strength test and SPPB score.

## Results

### Demographic characteristics

Forty-five women with fibromyalgia and 39 healthy control female subjects were included in our study. Their demographic and clinical characteristics are represented in Table 1.

**Table 1:** Demographic and clinical characteristics of the population including two groups, fibromyalgia and controls, represented by means ± standard deviation and adjusted *p* value

	Fibromyalgia, *N* = 45	Controls, *N* = 39	Adjusted *p* value
Age (years)	48.86 ± 8.66	44.35 ± 7.29	**0.013**
Weight (kg)	68.88 ± 10.86	62.92 ± 11.83	**0.019**
Height (m)	1.61 ± 0.06	1.64 ± 0.06	0.06
BMI (kg/m^2^)	26.24 ± 3.26	23.13 ± 3.53	** < 0.001**

*BMI* Body mass index

### Assessment of sarcopenia in the different study groups

Table 2 shows the variables to assess sarcopenia for the two groups.

**Table 2:** Variables used to assess sarcopenia in the two groups, with means ± standard deviation and adjusted *p* value

Variable	Fibromyalgia (*n* = 45)	Control (*n* = 39)	Adjusted *p* value
FIQ (/100)	74 ± 13	15 ± 13	** < 0.001**
SARC-F (/10)	5 ± 2	0 ± 0	** < 0.001**
Fat mass (kg)	23.8 ± 7.1	23.4 ± 7.7	0.217
Lean mass (kg)	42.4 ± 6.3	42.8 ± 6.4	0.304
SMM (kg)	19.2 ± 2.7	19.6 ± 2.8	0.260
SMI (kg/m^2^)	7.2 ± 0.5	7.4 ± 0.7	0.263
Handgrip strength test (kg)	18 ± 8	30 ± 6	** < 0.001**
SPPB score (/12)	8 ± 2	12 ± 0	** < 0.001**

*SMM* skeletal muscle mass, *SMI* skeletal muscle mass index, *SPPB* Short Physical Performance Battery

#### FIQ and SARC-F questionnaires

The mean value of the FIQ questionnaire for the group with fibromyalgia (73 ± 13) is significantly higher than that of the control group (15 ± 13) (*p* < 0.001), displaying severe disease.

The FIQ value is not correlated with the physical performance (coefficient correlation = − 0.28, *p* = 0.062), nor with the muscle strength (coefficient correlation = − 0.23, *p* = 0.125). Therefore, the severity of the disease is not correlated with the muscle function.

The mean value of the SARC-F questionnaire was significantly higher (5 ± 2) in the fibromyalgia group compared to the control group (0 ± 0) (*p* < 0.001). SARC-F questionnaire is predictive of sarcopenia in patients with fibromyalgia.

#### Muscle mass

According to the EWGSOP, low muscle mass with the BIA for women is defined by SMI < 6.42 kg/m^2^. We did not observe any statistical difference between fibromyalgia and control groups regarding skeletal muscle mass (*p* = 0.263). There was no statistical difference for the fat mass (*p* = 0.217), lean mass (*p* = 0.304) and the SMM (*p* = 0.260).

#### Muscle strength

The cut-off value for the dominant hand in women, according to the EWGSOP, is below 20 kg. There was a statistically significant loss of muscle strength in fibromyalgia group (18 ± 8) compared to the control group (30 ± 6) (*p* < 0.001).

#### Physical performance

A SPPB score below 8 signs a loss of physical performance. Patients with fibromyalgia had a statistically significant lower SPPB score (8 ± 2) compared to control groups (12 ± 0) (*p* < 0.001). This shows that fibromyalgia patients have a loss of physical performance relative to controls.

## Discussion

The goal of this study was to determine whether the reduction of muscle function in fibromyalgia is related to sarcopenia according to the EWGSOP 2010 criteria. We have identified a loss of muscle function in fibromyalgia (loss of muscle strength and physical performance), but there was no loss of muscle mass, the key feature in sarcopenia.

Muscle mass was studied with a bio-electrical impedance analysis (BIA). Other methods are also used in clinical practice. For example, Magnetic Resonance Imaging (MRI) or the CT-scan are used for the non-invasive assessment of muscle mass. Dual-energy x-ray absorptiometry (DXA) is an alternative method for distinguishing the fat mass, the lean mass and bone density [24]. We chose the BIA because this method is not expensive and easy to use in daily practice [14]. The disadvantage of the BIA is that it overestimates muscle mass and underestimates fat mass [18]. Koca et al. assessed patients with fibromyalgia in terms of sarcopenia using BIA, anthropometrics measures, a handgrip strength test and a gait speed test over 6 m. Muscle strength and gait speed were lower in patients with fibromyalgia. The body composition according to the BIA and anthropometrics measures were not significantly different between the two groups [25].

The contribution of our study in relation to Koca’s work was to use the EWGSOP 2010 criteria and their threshold values for each component of sarcopenia. Physical performance was evaluated with different tests including a gait speed, a balance test and a chair stand test. Furthermore, a screening test of sarcopenia was realized, using the SARC-F questionnaire. Furthermore, we limited the age of all participants between 30 and 60 years because of the impending loss of muscle mass after the third decade [26]. BMI < 30 kg/m^2^ was included to uniform the two groups for the muscle mass, and to avoid the bias of significantly higher fat mass in obese patients that could warp the interpretation of the results.

Muscle mass of patients with fibromyalgia has been studied in different works and did not show any difference compared to healthy people, as assessed by BIA [11, 16, 25]. However, some studies showed a difference in body composition in terms of fat mass and lean mass, showing that women with fibromyalgia had more fat mass than healthy controls [11, 12]. In our study, we did not observe that body fat composition was different between the two study groups.

Regarding muscle function, we also observed similarly to other studies that patients with fibromyalgia exhibit a loss of muscle strength and physical performance [8–10, 13]. Loss of muscle function without loss of muscle mass is named dynapenia. It is defined as the age-related loss of muscle strength and it is often confused with sarcopenia [27]. Sarcopenia is the atrophy of muscular fibers associated with the reduction of the number of the fibers, while dynapenia is a muscular atrophy with a conserved number of fibers responsible for a loss of muscle strength without loss of muscle mass. Based on our results, we can define patients with fibromyalgia as suffering from dynapenia.

In early 2018, the EWGSOP updated the definition of sarcopenia with new recommendations. A low muscle strength is now considered to be the primary indicator of probable sarcopenia. When a low muscle strength is detected, a sarcopenia diagnosis must be confirmed by the presence of a concomitant low muscle mass. The severity of the disease is evaluated with the study of physical performance [15].

In clinical practice, according to the new recommendations in 2018 by the EWGSOP, the SARC-F questionnaire helps to find patients at risk of sarcopenia. A SARC-F score ≥ 4/10 is an indication to measure muscle strength. Low muscle strength is predictive of probable sarcopenia that must be confirmed with the measure of low muscle mass. Low physical performance with a SPPB score ≤ 8/12 indicates the severity of the sarcopenia. According to those criteria, sarcopenia is now considered as a muscle disease. Otherwise, cut-off points have been reviewed for muscle strength (< 16 kg for women) and data are available for men and women according to their centiles. If we apply the new recommendations, the SARC-F questionnaire is predictive of sarcopenia in our Fibromyalgia population. The mean muscle strength in this group is 18 kg, but according to the mean age of 48 years old, we are under the 10th centile, defining low muscle strength and probable sarcopenia. As muscle mass was normal using the BIA, which is known to overestimate muscle mass, we cannot confirm the disease.

Some limitations should be considered in our study. First, we did not consider the sedentary lifestyle of the controls, the hormonal or the nutritional status, nor comorbidities. Moreover, alcohol consumption, smoking or drug uses were not taken into account in our study. Second, assessment of sarcopenia was performed during a single visit and not repeatedly over a time span for more accurate determination of muscle mass loss.

Finally, we decided to consider only women in our study because FM is mostly a women’s disorder [28].

## Conclusions

There is no sarcopenia in patients with fibromyalgia according to the original definition of the EWGSOP in 2010, but there is a possible sarcopenia with the 2018 updates of the algorithm. Our study demonstrated a significant reduction in muscle function in fibromyalgia patients (decreased in muscle strength and in physical performance) without any loss of muscle mass. Loss of muscle function without decrease in muscle mass is named dynapenia. Screening to detect and prevent sarcopenia should be conducted in patients with fibromyalgia as loss of muscle function is common in these patients.

## Supplementary Information

Below is the link to the electronic supplementary material.

Supplementary file1 (DOCX 17 KB)
