# Supplementary material for: Assessment of sarcopenia in patients with fibromyalgia
Source: Rheumatol Int. 2021 Aug 21;42(2):279–84. doi: 10.1007/s00296-021-04973-6 (PMC8800887; doi:10.1007/s00296-021-04973-6)
Supplement: Supplementary file 1 — Supplementary file1 (DOCX 17 KB) [file 296_2021_4973_MOESM1_ESM.docx]

**Assessment of sarcopenia in patients with fibromyalgia: complementary data**

## ***Propensity score***

Propensity score was obtained: the first part of the table (“Before Matching”) reports the means and standard deviations for data in the two groups (Fibromyalgia, Control) as well as standardized difference, and indicates that, in almost all cases, the two groups have different means for the different variables before matching; that is, the standardized difference is large. The second part of the table presents the results after matching. The standardized difference values indicate that the two groups have now similar means for the different variables after matching; that is, the absolute standardized difference is lower or equal to 10-15. Based on this matching table, we considered the Fibromyalgia and Control groups similar on covariates chosen for the propensity score.

Propensity score obtained for the two groups. This table represents data before and after matching with means and standard deviation; the ASD that equal to 0 after matching indicate that the two groups are similar on covariates chosen for the propensity score.

|  | Before Matching | | | After Matching | | |
| --- | --- | --- | --- | --- | --- | --- |
| Variables | ***Fibromyalgia (n=45)*** | ***Control (n=39)*** | ***ASD*** | ***Fibromyalgia (n=45)*** | ***Control (n=39)*** | ***ASD*** |
| *Age* | 48,86 (8,66) | 44,35 (7,29) | 56,29% | 47,18 (7,84) | 47,18 (8,9) | 0,00% |
| *Weight* | 68,88 (10,86) | 62,92 (11,83) | 52,50% | 66,16 (11,68) | 66,16 (12,25) | 0,00% |
| *Size* | 1,61 (0,06) | 1,64 (0,06) | 41,72% | 1,62 (0,07) | 1,62 (0,07) | 0,00% |
| *BMI* | 26,24 (3,26) | 23,13 (3,53) | 91,24% | 24,84 (3,61) | 24,84 (3,57) | 0,00% |

ASD: Absolute Standardized Difference
